# Role of Protein Farnesylation in Burn-Induced Metabolic Derangements and Insulin Resistance in Mouse Skeletal Muscle

**DOI:** 10.1371/journal.pone.0116633

**Published:** 2015-01-16

**Authors:** Harumasa Nakazawa, Marina Yamada, Tomokazu Tanaka, Joshua Kramer, Yong-Ming Yu, Alan J. Fischman, J. A. Jeevendra Martyn, Ronald G. Tompkins, Masao Kaneki

**Affiliations:** 1 Department of Anesthesia, Critical Care and Pain Medicine, Massachusetts General Hospital, Harvard Medical School, Charlestown, Massachusetts, United States of America; 2 Shriners Hospitals for Children, Boston, Massachusetts, United States of America; 3 Department of Pathology, Massachusetts General Hospital, Harvard Medical School, Boston, Massachusetts, United States of America; 4 Department of Surgery, Massachusetts General Hospital, Harvard Medical School, Boston, Massachusetts, United States of America; Virginia Commonwealth University, UNITED STATES

## Abstract

**Objective:**

Metabolic derangements, including insulin resistance and hyperlactatemia, are a major complication of major trauma (e.g., burn injury) and affect the prognosis of burn patients. Protein farnesylation, a posttranslational lipid modification of cysteine residues, has been emerging as a potential component of inflammatory response in sepsis. However, farnesylation has not yet been studied in major trauma. To study a role of farnesylation in burn-induced metabolic aberration, we examined the effects of farnesyltransferase (FTase) inhibitor, FTI-277, on burn-induced insulin resistance and metabolic alterations in mouse skeletal muscle.

**Methods:**

A full thickness burn (30% total body surface area) was produced under anesthesia in male C57BL/6 mice at 8 weeks of age. After the mice were treated with FTI-277 (5 mg/kg/day, IP) or vehicle for 3 days, muscle insulin signaling, metabolic alterations and inflammatory gene expression were evaluated.

**Results:**

Burn increased FTase expression and farnesylated proteins in mouse muscle compared with sham-burn at 3 days after burn. Simultaneously, insulin-stimulated phosphorylation of insulin receptor (IR), insulin receptor substrate (IRS)-1, Akt and GSK-3β was decreased. Protein expression of PTP-1B (a negative regulator of IR-IRS-1 signaling), PTEN (a negative regulator of Akt-mediated signaling), protein degradation and lactate release by muscle, and plasma lactate levels were increased by burn. Burn-induced impaired insulin signaling and metabolic dysfunction were associated with increased inflammatory gene expression. These burn-induced alterations were reversed or ameliorated by FTI-277.

**Conclusions:**

Our data demonstrate that burn increased FTase expression and protein farnesylation along with insulin resistance, metabolic alterations and inflammatory response in mouse skeletal muscle, all of which were prevented by FTI-277 treatment. These results indicate that increased protein farnesylation plays a pivotal role in burn-induced metabolic dysfunction and inflammatory response. Our study identifies FTase as a novel potential molecular target to reverse or ameliorate metabolic derangements in burn patients.

## Introduction

Stress-associated metabolic derangements in skeletal muscle are a major complication of major trauma, including severe burn injury, and affect the long-term outcome of burn patients [1,2]. These metabolic aberrations include hypermetabolism, catabolism, insulin resistance, hyperlactatemia, and muscle wasting [3–5]. Hyperlactatemia is an early predictor of the mortality of burn patients [6–8]. Insulin resistance has been considered as a major common denominator of these metabolic alterations under stress condition, including burn injury.

Strict glycemic control by intensive insulin therapy has been implemented in some intensive care units since the beneficial effects on the mortality and prognosis were reported [9]. Recently, however, risk of hypoglycemia during the intensive insulin therapy has emerged as a problem in critical care [10,11]. Moreover, insulin sensitizers in critically ill patients pose several drawbacks and limitations. The adverse cardiovascular side effects of thiazolidinediones [12,13] and metformin-related lactic acidosis [14] may limit use of these insulin sensitizers in critically ill patients, such as burn patients. These clinical scenarios have urged us to further investigate the molecular mechanisms underlying burn-induced insulin resistance and metabolic derangements with the goal of identifying (a) molecular target(s) to reverse metabolic dysfunction of patients with major trauma (e.g., burn injury) [15].

The insulin receptor (IR)-insulin receptor substrates (IRSs)-Akt pathway plays a central role in metabolic actions of insulin. IRS-1 plays a pivotal role in metabolic actions of insulin in skeletal muscle, while IRS-2 has a more prominent role than IRS-1 in liver metabolism [16]. Akt is activated by phosphorylation of threonine 308 and serine 473. Akt phosphorylates GSK-3β at serine 9, leading to inhibition of GSK-3β activity. GSK-3β inhibits glycogen synthesis. Attenuated Akt activity, therefore, results in increased GSK-3β activity, which, in turn, leads to decreased glycogen synthesis. Muscle-specific insulin receptor knockout mice do not exhibit hyperglycemia or hyperinsulinemia although Akt-mediated insulin signaling is abolished in skeletal muscle [17]. Defective insulin signaling in skeletal muscle causes hyperlactatemia, and decreased glycogen content and increased protein degradation in muscle [18,19]. These findings suggest that impaired insulin signaling in skeletal muscle can cause metabolic alterations of lactate, glycogen and proteolysis independent of hyperglycemia or hyperinsulinemia.

Protein-tyrosine phosphatase (PTP)-1B downregulates insulin signaling by dephoshorylating tyrosine residues in IR and IRS-1 [20]. Phosphatase and Tensin Homolog Deleted from Chromosome 10 (PTEN) inhibits insulin-stimulated phosphorylation (activation) of Akt at threonine 308 and serine 473 by dephosphorylating phosphatidylinositol 3, 4, 5- triphosphate, a product of phosphatidylinositol 3-kinase [21].

Protein farnesylation is a lipid modification of the cysteine residues in the CAAX motif located in the carboxyl terminus of proteins (“C” is cysteine, “A” is aliphatic amino acid, and “X” is any amino acid at the carboxyl terminus, but usually serine, methionine, glutamine, or alanine). Farnesyltransferase (FTase) catalyzes the covalent attachment of farnesyl pyrophosphate via a thioester linkage to cysteines in the CAAX box. It has been established that farnesylation plays important roles in maturation, activity, and membrane localization of some proteins, including the Ras family small G-proteins, under basal (physiological) condition. On the other hand, limited knowledge is available about the role of protein farnesylation in the pathological conditions, including major trauma. We have previously shown that farnesylated proteins and FTase activity increase in spleen of septic mice compared with sham mice [22], and that a competitive inhibitor for FTase, FTI-277, improves survival after induction of sepsis or lipopolysaccharide (LPS) challenge in mice [22,23]. FTI-277 prevents systemic inflammatory response, as indicated by reversal of increased circulating high-mobility group box 1 (HMGB1) and histone H3 levels. It is noteworthy that amelioration of inflammatory response parallels the reversal of increased FTase activity as well as increased farnesylated proteins content in septic mice [22]. These results raise the possibility that increased farnesylation may act as an upstream enhancer of inflammatory response as well as a downstream mediator, functioning as an integral component of inflammatory response, which, in turn, plays a pivotal role in the development of insulin resistance and metabolic alterations. This possibility, however, remains an open question.

The potential anti-inflammatory action of farnesylation inhibition has been postulated by the accumulated evidence of the cholesterol-lowering-independent beneficial effects of statins, inhibitors of 3-hydroxy-3-methylglutaryl coenzyme A (HMG-CoA) reductase. Statins reduce the biosynthesis of farnesyl pyrophosphate, which is a precursor of cholesterol and geranylgeranyl pyrophosphate, as well as a substrate of farnesylation. It has been proposed, therefore, that inhibition of protein isoprenylation, namely farnesylation and geranylgeranylation, may mediate the lipid-lowering-independent pleiotropic effects of statins, including anti-inflammatory action, although direct evidence is lacking.

In a previous study in elderly burn patients, preinjury statin use was associated with 83% reduction in the odds of death after burn injury [24]. Similarly, we have previously shown in mice that simvastatin ameliorates burn-induced glucose intolerance [25] and improves survival after post-burn sepsis [26]. In line with this, a previous study has shown that atorvastatin improves survival and insulin signaling in tissues, including skeletal muscle, of septic rats [27]. Nonetheless, the molecular mechanisms underlying the insulin-sensitizing effect of statins remain largely unknown.

Inflammatory response plays a crucial role in obesity- and stress-induced insulin resistance [28–30]. Collectively, one can speculate that statin may reverse or ameliorate stress-induced insulin resistance by attenuating inflammatory response via inhibition of protein isoprenylation. However, the effects of FTase inhibition on insulin signaling or metabolic alterations, which are associated with local and systemic inflammatory response, have not yet been studied. These findings motivated us to test the hypothesis that inhibition of increased protein farnesylation may reverse or ameliorate burn-induced muscle insulin resistance and metabolic alterations along with mitigation of inflammatory response. We studied, therefore, the effects of a specific inhibitor of FTase, FTI-277, in burned mice.

## Methods

### Ethics Statement

All experiments were carried out in accordance with the institutional guidelines and the study protocol was approved by the Institutional Animal Care and Use Committee (IACUC) at the Massachusetts General Hospital (the protocol title: Stress-Associated Insulin Resistance; the protocol#: 2007N000020). The animal care facility is accredited by the Association for Assessment and Accreditation of Laboratory Animal Care.

### Animals

We used male C57BL/6 mice (Jackson Laboratory, Bar Harbor, ME) at 8 weeks of age. The mice were housed in a pathogen-free animal facility with 12 h light/dark cycles at 25°C. A full-thickness burn injury comprising 30% of total body surface area was produced under anesthesia with pentobarbital sodium (50 mg/kg BW/day, IP) in mice by immersing the abdomen for 6 sec and both sides of the flank for 4 sec in 80°C water. We confirmed this procedure produced full-thickness burn injury in mice by hematoxylin and eosin staining. Sham-burned mice were immersed in lukewarm water. Buprenorphine (0.1 mg/kg BW, SC) was administered every 8 h up to 48 h after burn or sham-burn. For resuscitation, prewarmed normal saline (0.04 ml/g BW, IP) was injected just after burn or sham-burn regardless of the treatment. Starting at 2 h after burn or sham-burn, the mice were treated with FTI-277 (N-[4-[2(R)-amino-3-mercaptopropyl]amino-2-phenylbenzoyl]methionine methyl ester trifluoroacetate salt) (5 mg/kg BW/day, IP, Sigma, St. Louis, MO) or vehicle (phosphate-buffered saline [PBS]) for 3 days.

### Tissue Homogenization and Immunoblotting

At 3 days after burn or sham-burn, following an overnight fasting, the mice received insulin (0.3 U/kg BW, Humulin R, Eli Lilly) or saline via the portal vein under anesthesia with pentobarbital sodium (50 mg/kg BW, IP), and rectus abdominis was collected at 5 min thereafter [31,32]. The muscle samples were snap-frozen and kept at −80°C until biochemical analyses were performed. The samples were homogenized as described previously [33]. Briefly, tissues were pulverized under liquid nitrogen and homogenized in homogenization buffer (50 mM HEPES, pH 8.0, 150 mM NaCl, 2 mM EDTA, 7.5% lithium dodecylsulfate, 2% CHAPS, 10% glycerol, 10 mM sodium fluoride, 2 mM sodium vanadate, 1 mM PMSF, 10 mM sodium pyrophosphate, 1 mM DTT, protease inhibitor cocktail [Sigma]). After incubation for 30 min, the homogenized samples were centrifuged at 14,000 rpm for 10 min. Equal amounts of protein were subjected to a standard SDS–polyacrylamide gel electrophoresis and were electrophoretically transferred to a nitrocellulose membrane (Bio-Rad, Hercules, CA). The equal protein loading was confirmed by Ponceau S staining. The membranes were soaked in blocking buffer (GE Healthcare, Pittsburgh, PA) for 1 h and then incubated overnight at 4°C with anti-glyceraldehyde 3-phosphate dehydrogenase (GAPDH) (Trevigen, Gaithersburg, MD, #2275-PC-1), anti-Akt (#4691), anti-phospho-Akt at threonine 308 (#2965), anti-phospho-Akt at serine 473 (#4058), PTEN (#9552), anti-phospho-PTEN at serine 380 (#9551), anti-phospho-GSK-3β at serine 9 (#9336) (Cell Signaling Technology, Danvers, MA), anti-phospho-IR at tyrosine 972 (44–800G), anti-phospho IRS-1 at tyrosine 612 (IRS-1) (44–816G) (Life Technologies, Grand Island, NY), anti-IR (07–724), IRS-1 (06–248), PTP-1B (07–088) (Millipore, Billerica, MA), anti-GSK-3β (BD Bioscience, San Jose, CA, #610202), or anti-farnesyltansferase (Santa Cruz Biotechnology, Santa Cruz, CA, #sc-137), followed by incubation with HRP-conjugated anti-rabbit IgG or anti-mouse IgG antibody (GE Healthcare) for 1 h at room temperature. Immunoreactive bands were detected with ECL Advance Western Blotting Detection Kit (GE Healthcare) and scanned using the HP Scanjet 4850 (Hewlett-Packard, Palo Alto, CA). Densitometric analysis of the results was carried out using NIH Image software (ver. 1.62). Protein expression was normalized to GAPDH unless otherwise indicated.

### Measurement of Glycogen Content

Muscle glycogen content was determined using anthrone reagent as previously described [33]. Muscle was solubilized in 0.3 ml of 30% (w/v) KOH solution at 95°C for 30 min with vortexing every 10 min. Then, 0.1 ml of 1 m Na_2_SO_4_ and 0.8 ml of 100% ethanol were added to the sample. The mixture was centrifuged at 14,000 rpm for 5 min. After washing twice with 1 ml of 70% ethanol, the precipitate was dissolve in 0.4 ml of water. One milliliter of anthrone reagent (66% H_2_SO_4_, 0.05% anthrone, 1% thiourea) was added to 0.2 ml of the sample, and the mixture was boiled for 15 min. The concentrations of glycogen were determined by measuring absorbance at 620 nm and normalized to the tissue weight.

### Measurement of Triglyceride Content

At 3 days after burn or sham-burn, muscle samples were collected under anesthesia after 4h fasting. Muscles were hydrolyzed in 5% (w/v) Triton X-100 solution, and boiled for 5 min. Muscle triglyceride content were determined using a commercial kit (Sigma) according to the manufacture’s instructions and normalized to the tissue weight [33].

### Measurement of Amino Acids and Lactate Release during *Ex vivo* Muscle Incubation

Harvested muscle was incubated for 2 h in Krebs-Henseleit bicarbonate (KHB) buffer (pH 7.4) (120 mM NaCl, 4.8 mM KCl, 25 mM NaHCO_3_, 2.5 mM CaCl_2_, 1.2 mM KH_2_PO_4_, and 1.2 mM MgSO_4_) supplemented with 5 mM HEPES and 0.1% fatty acid-free bovine serum albumin containing 5 mM glucose and 0.5 mM cycloheximide, the latter of which inhibits protein synthesis and therefore blocks re-incorporation of released amino acids into proteins, as previously described [34]. After 2-h incubation, L-lactate and L-amino acids in the incubation buffer were measured using commercial kits (BioVision, Mountain view, CA) according to the manufacturer’s instructions and normalized to the tissue weight.

### Measurement of Insulin-stimulated Glucose Uptake During *Ex vivo* Muscle Incubation

Glucose uptake was measured as previously described [32]. Briefly, at 3 days after burn or sham-burn, mice were anesthetized following an overnight fasting, and rectus abdominis were dissected and rapidly split by mid-incision into two muscle strips. After the muscle strips were rinsed briefly in KHB buffer (pH 7.4) supplemented with 32 mM mannitol, they were incubated in 5 mL of KHB buffer supplemented with 8 mM glucose and 32 mM mannnitol in the presence or absence of insulin (2 mU/mL, Humulin R, Eli Lily, Indianapolis, IN) in a shaking water bath at 37°C for 20 min. Next, the muscles were incubated for 20 min at 37°C in 2 mL of KHB buffer containing 2-deoxy-[3H] glucose (2.5 μCi/mL, PerkinElmer, Wltham, MA) and [14C] mannitol (0.3 μCi/mL,PerkinElmer) with or without insulin in a shaking incubator. Buffers were gassed continuously with 95% O2: 5% CO2 throughout the experiment. The muscles were then rinsed with KHB buffer, rapidly blotted, weighed, and solubilized by incubation at 60°C for 1 h in 0.5 mL of 1N NaOH. Radioactivity in the sample was counted using a scintillation counter. 2-Deoxy-[3H] glucose uptake rates were corrected for extracellular trapping using [14C] mannitol counts and normalized to the tissue weight. Insulin-stimulated glucose uptake was calculated based on differences in glucose uptake in the presence and absence of insulin.

### Evaluation of mRNA Expression Levels

Total RNA was purified using TRIzol reagent (Life Technologies, Grand Island, NY). The first-strand cDNA was synthesized from 1 μg of total RNA using a high capacity cDNA reverse transcription kit (Life Technologies). Real-time PCR reactions were performed using 10 ng of cDNA and TaqMan probes (Life Technologies) for inducible nitric oxide synthase (iNOS) (Mm00440502_m1), interleukin-1β (IL-1β) (Mm99999061_mH), tumor necrosis factor-α (TNF-α) (Mm00443258_m1), toll-like receptor-4 (TLR-4) (Mm00445274_m1), cycloxygenase- 2 (COX-2) (Mm00478374_m1), PTP-1B (Mm00448427_m1), PTEN (Mm00477208_m1), 18S ribosomal RNA (Hs99999901_s1) and GAPDH (Mm99999915_g1) using Mastercycler (Eppendorf, Westbury, NY). 36B4 mRNA levels were measured using SYBR Green Dye (Life Technologies) and specific primers (Forward: 5’-GAAGACAGGGCGACCTGGAA-3’; Reverse: 5’-TTGTCTGCTCCCACAATGAAGC-3’) [35]. mRNA levels were normalized to the geometric mean of three internal control genes, 18S ribosomal RNA, GAPDH and 36B4, as previously described [36]. The geometrical average of the three internal controls did not differ between the groups.

### Measurement of Farnesylated Proteins

The amounts of farnesylated proteins in the samples were measured as previously described [22] with minor modifications. Briefly, the muscle was lysed in lysis buffer (50 mM HEPES, pH 8.0, 150 mM NaCl, 10 mM sodium pyrophosphate, 10 mM NaF, 1 mM PMSF, 10% glycerol, 2 mM EDTA, 2 mM sodium vanadate, 1 mM DTT, 1% Nonidet P-40, 0.1% SDS, protease inhibitor cocktail). The protein concentration was measured by BCA kit (Thermo Fisher Scientific, Waltham, MA). After 96-well plates were coated with the lysates (150 μg/well) at 4°C overnight, the samples were incubated with anti-farnesylated cysteine antibody (0.25 μg/well) (abcam, Cambridge, MA, #ab12432) at room temperature for 2 h, washed with PBS containing 0.05% Tween 20, and incubated with biotin-conjugated anti-rabbit IgG antibody (Sigma, #B8895) at room temperature for 2 h. After washing with PBS containing 0.05% Tween 20, they were incubated with extravidin-alkaline phosphatase (Sigma, #E2636) at room temperature for 1 h, followed by washing with PBS containing 0.05% Tween 20 and the addition of the substrate of alkaline phosphatase, PNPP (p-nitrophenyl phosphate disodium salt, Sigma) diluted in diethanolamine. After 1-h incubation with PNPP, the absorbance at 415 nm was measured by a microplate reader.

### Measurement of plasma lactate, HMGB1 and histone H3 concentrations

L-lactate concentrations in heparinized plasma were measured using a commercial kit (BioVision). HMGB1 and histone H3 concentrations were measure at 3 days after burn or sham-burn using ELISA kits from Shino-Test Corporation (Tokyo, Japan) and Cell Signaling Technology, respectively, according to the manufactures’ instructions.

### Statistical Analysis

To analyze the effects of burn injury at different time points, the data were compared with one-way ANOVA followed by Tukey multiple comparison test. To analyze the effects of FTI-277 in burned and sham-burned mice, two-way ANOVA followed by Tukey multiple comparison test was used. A value of p<0.05 was considered statistically significant. All values are expressed as means ± SEM.

## Results

### Increases in FTase, PTP-1B and PTEN expression in muscle were associated with hyperlactatemia after burn injury in mice

**Figure 1:**
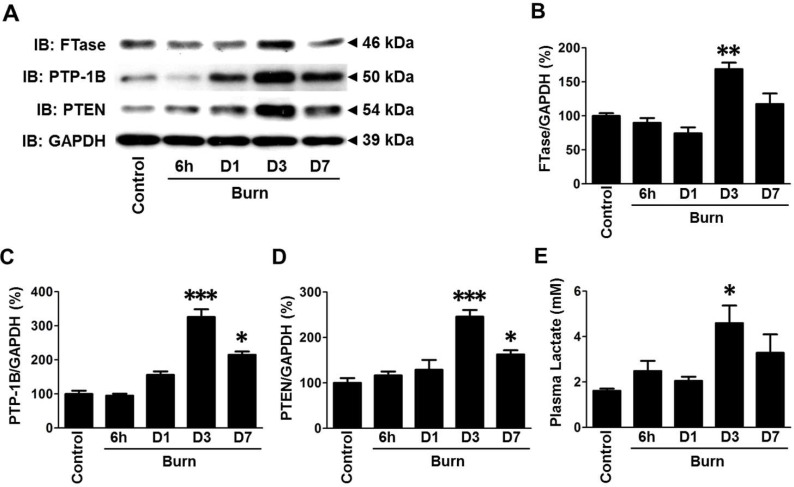
Increases in farnesyltransferase (FTase), PTP-1B and PTEN expression were associated with hyperlactatemia following burn injury in mice. Muscle and blood samples were collected from naïve mice (Control), and at 6 h, 1 (D1), 3 (D3) and 7 days (D7) after burn injury. Immunoblot analysis revealed that FTase protein expression was significantly increased at 3 days after burn compared with naïve mice (B). PTP-1B and PTEN protein expression were significantly increased at 3 days after burn compared with naïve mice (C, D). FTase, PTP-1B and PTEN expression were normalized to that of GAPDH. Plasma lactate concentration was significantly increased at 3 days after burn compared with naïve mice (E). *p<0.05, **p<0.01, ***p<0.001 vs. Control. n = 4 mice at each time point.

Burn injury increased FTase protein expression in skeletal muscle at 3 days after burn (Fig. 1A, 1B), where FTase expression in burned mice was increased to 169% of that in naïve mice (p<0.01). On the other hand, GAPDH expression was not altered by burn (S1A Fig.).

Similar to the pattern of FTase expression, protein expression of PTP-1B and PTEN, negative regulators of insulin signaling, increased in skeletal muscle after burn in a time-dependent manner and the maximum levels were observed at 3 days after burn (Fig. 1A, 1C, 1D). Likewise, plasma lactate levels were increased after burn in a time-dependent manner and the maximum plasma lactate level was observed at 3 days after burn (Fig. 1E). At 3 days after burn, plasma lactate level was increased to 285% of that in naïve mice (p<0.05).

### FTI-277 treatment reversed burn-induced impaired insulin signaling in mouse skeletal muscle

**Figure 2:**
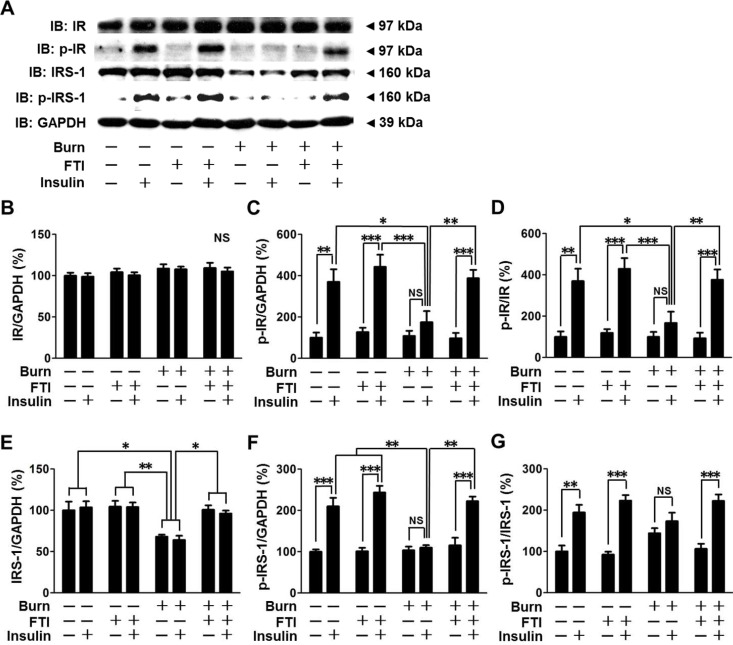
FTI-277 treatment reversed burn-induced impaired insulin receptor (IR)-insulin receptor substrate-1 (IRS-1) signaling in skeletal muscle. IR protein expression was not altered by burn, FTI-277 or insulin (B). Insulin-stimulated IR phosphorylation was attenuated at 3 days after burn compared with sham-burned mice, which was almost completely restored by FTI-277 treatment (C, D). Burn decreased IRS-1 protein expression (E) and insulin-stimulated IRS-1 phosphorylation (F, G), both of which were reversed by FTI-277. Insulin significantly increased p-IRS-1/GAPDH ratio and p-IRS-1/IRS-1 ratio in sham-burned mice and FTI-277-treated burned mice, whereas insulin failed to significantly increase p-IRS-1/GAPDH ratio or p-IRS-1/IRS-1 ratio in vehicle-treated burned mice (F, G). There was a trend of increase in insulin-stimulated p-IRS-1/IRS-1 ratio in vehicle-treated burned mice, but there was no statistical difference (G). n = 5 mice per group for saline-injected mice, n = 6 mice per group for insulin-injected sham-burned mice, n = 8 mice per group for insulin-injected burned mice. *p<0.05, **p<0.01, ***p<0.001, NS: not significant.

The maximum effects of burn on FTase, PTP-1B and PTEN expression and plasma lactate levels were observed at 3 days after burn (Fig. 1). Similarly, our previous study in mice has shown that burn-induced impairment in muscle insulin signaling is most prominent at 3 days after burn [32]. We examined, therefore, the effects of FTI-277 on insulin signaling at 3 days after burn. When treated with vehicle alone, burn impaired insulin-stimulated phosphorylation (activation) of IR and IRS-1, in skeletal muscle compared with vehicle-treated sham-burned mice (Fig. 2). In vehicle-treated burned mice, insulin failed to significantly increase phosphorylation of IR and IRS-1 compared with saline, although there was a trend of insulin-stimulated increase in phosphorylation of IR. Impaired insulin-stimulated phosphorylation of IR and IRS-1 in burned mice was reversed by FTI-277. Protein expression of IR and GAPDH was not altered by burn, FTI-277 or insulin (Fig. 2A, 2B, S1B). In contrast, IRS-1 protein expression was significantly decreased by burn, in agreement with previous studies [32,37]. Burn-induced suppressed IRS-1 protein expression was restored by FTI-277 (Fig. 2E). When phosphorylated IRS-1 was normalized to total IRS-1 protein expression, p-IRS-1/IRS-1 ratio did not significantly differ between sham-burned and burned mice. It is important to note that insulin failed to significantly increase p-IRS-1/IRS-1 ratio in vehicle-treated burned mice, whereas p-IRS-1/IRS-1 ratio was markedly increased by insulin in sham-burned mice and FTI-277-treated burned mice (Fig. 2G).

**Figure 3:**
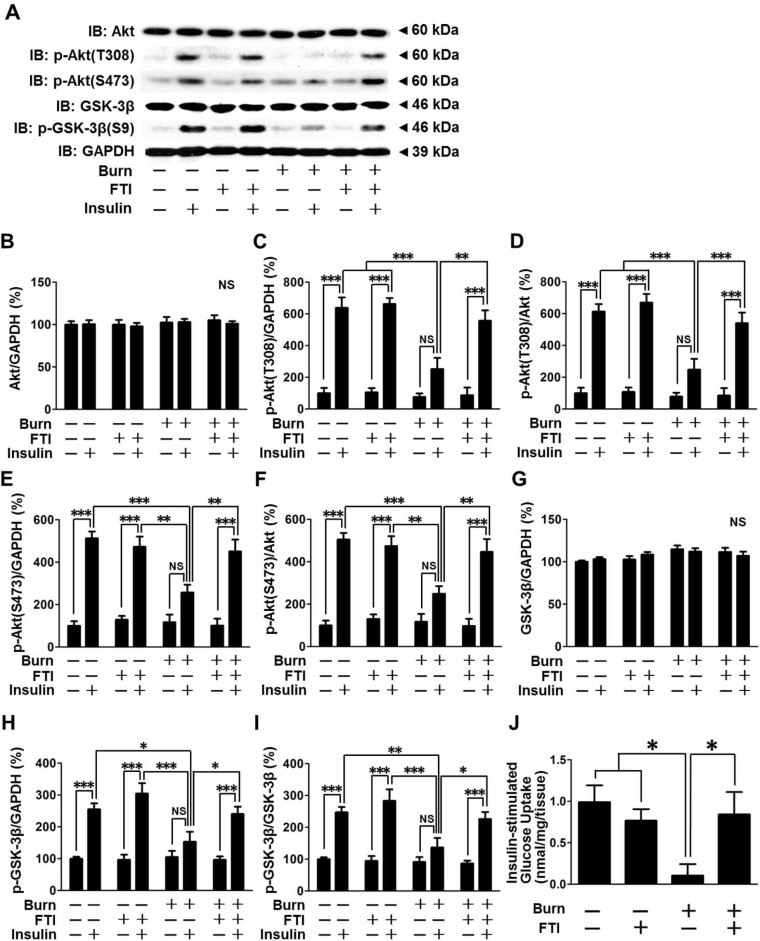
FTI-277 treatment reversed burn-induced attenuated insulin-stimulated phosphorylation of Akt and GSK-3β, and glucose uptake in skeletal muscle. Protein expression of Akt and GSK-3β was not altered by burn, FTI-277 or insulin (B, G). Insulin-stimulated phosphorylation of Akt at threonine 308 and serine 473 was blunted at 3 days after burn compared with sham-burn, which was reversed by FTI-277 treatment (C-F). Likewise, insulin-stimulated phosphorylation of GSK-3β at serine 9 was decreased in burned mice, which was reversed by FTI-277 treatment (H, I). n = 5 mice per group for saline-injected mice, n = 6 mice per group for insulin-injected sham-burned mice, n = 8 mice per group for insulin-injected burned mice. (J) Insulin-stimulated glucose uptake was attenuated by burn injury, and FTI-277 restored it in burned mice. n = 4 mice per group for sham-burned mice, n = 6 mice per group for burned mice. *p<0.05, **p<0.01, ***p<0.001, NS: not significant.

Next, we examined the effects of FTI-277 on insulin-stimulated phosphorylation of Akt and GSK-3β in skeletal muscle of burned mice. Protein expression of Akt and GSK-3β was not altered by burn, FTI-277 or insulin (Fig. 3A, 3B, 3G). Insulin-stimulated phosphorylation of Akt at threonine 308 and serine 473 and GSK-3β at serine 9 was blunted in vehicle-treated burned mice, as compared with sham-burned mice (Fig. 3C, 3D, 3E, 3F, 3H, 3I). There was a trend of increased phosphorylation of Akt and GSK-3β by insulin in vehicle-treated burned mice compared with saline, but statistically significant differences were not found between insulin and saline injections in these animals. This contrasts to robust phosphorylation of Akt and GSK-3β in sham-burned mice. Decreased insulin-stimulated Akt and GSK-3β phosphorylation in burned mice was almost completely restored by FTI-277. In sham-burned mice, insulin-stimulated phosphorylation of GSK-3β appears to be increased by FTI-277 compared with vehicle, but there was no statistically significant difference.

Insulin-stimulated Akt phosphorylation plays a central role in insulin-stimulated glucose uptake [38]. We evaluated, therefore, insulin-stimulated glucose uptake in muscle *ex vivo*. Insulin-stimulated glucose uptake was significantly decreased by burn injury compared with sham-burn, which was restored by FTI-277 (Fig. 3J).

### FTI-277 treatment prevented burn-induced increased PTP-1B and PTEN expression in mouse skeletal muscle

**Figure 4:**
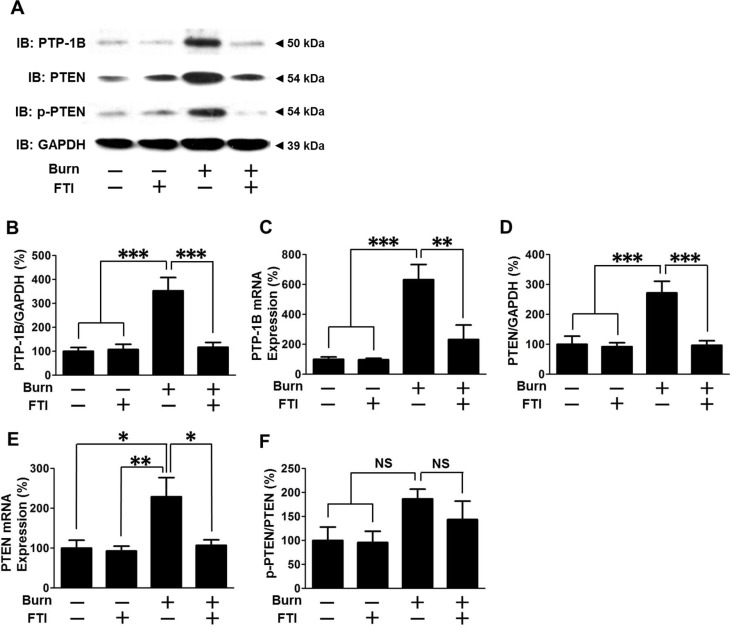
FTI-277 treatment inhibited burn-induced increased PTP-1B and PTEN expression in skeletal muscle. At 3 days after burn or sham-burn, PTP-1B protein expression (B) and mRNA expression (C) were increased in vehicle-treated burned mice compared with sham-burned mice. FTI-277 treatment significantly decreased PTP-1B protein and mRNA expression in burned, but not sham-burned, mice. Likewise, PTEN protein (D) and mRNA expression (E) were significantly increased in vehicle-treated burned mice, both of which were reversed by FTI-277. However, phosphorylated PTEN-to-total PTEN expression (p-PTEN/PTEN) ratio was not significantly altered by burn or FTI-277, although it appears to increase after burn injury (F). n = 6 mice per group. *p<0.05, **p<0.01, ***p<0.001, NS: not significant.

We examined the effects of burn and FTI-277 on expression of PTP-1B and PTEN, negative regulators of insulin signaling. Burn increased protein expression of PTP-1B and PTEN to 353% and 275% of those in sham-burned mice when treated with vehicle alone, respectively (p<0.001). FTI-277 treatment prevented increased PTP-1B and PTEN protein expression in burned mice as compared with vehicle alone (Fig. 4). In contrast, FTI-277 did not significantly alter protein expression of PTP-1B and PTEN in sham-burned mice. Burn significantly increased PTP-1B and PTEN mRNA expression in skeletal muscle, which was reversed by FTI-277 (Fig. 4C, 4E). On the other hand, GAPDH expression was not altered by burn and FTI-277 (S1C Fig.).

Phosphorylation of PTEN at serine 380 increases protein stability and thereby increases PTEN protein expression independent of transcription and translation [39,40]. Phosphorylation of PTEN at serine 380 was increased by burn injury when normalized to GAPDH expression, which was inhibited by FTI-277 (S2 Fig.). When phosphorylated PTEN was normalized to total PTEN expression, there was a trend of increased p-PTEN/PTEN ratio in burned mice, which appears to be partially inhibited by FTI-277. However, there was no statistically significant difference in p-PTEN/PTEN ratio. Together, one can speculate that the increases in mRNA level and phosphorylation at serine 380 may contribute in concert to the burn-induced increased PTEN expression.

### FTI-277 treatment reversed burn-induced metabolic alterations in mouse skeletal muscle

**Figure 5:**
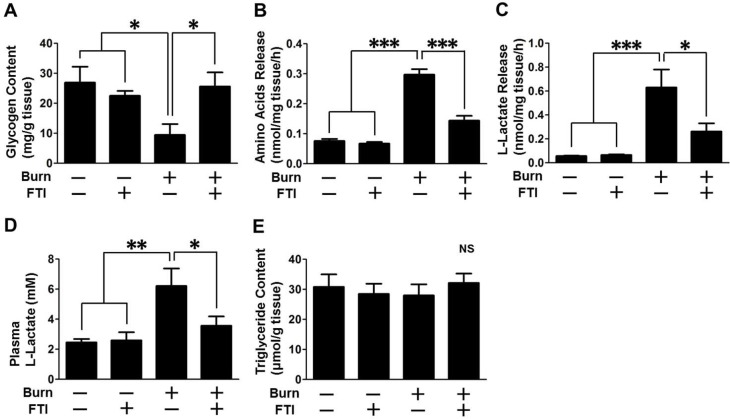
FTI-277 treatment ameliorated burn-induced metabolic alterations. Glycogen content was decreased in skeletal muscle at 3 days after burn compared with sham-burn. FTI-277 treatment prevented burn-induced reduction in glycogen content (A). *Ex vivo* release of amino acids (B) and lactate (C) from skeletal muscle was markedly increased by burn, both of which were mitigated by FTI-277 treatment. Plasma lactate level was increased by burn compared with sham-burn. FTI-277 treatment significantly inhibited burn-induced increase in plasma lactate level (D). Triglycerides content did not differ between the groups (E). n = 8 mice per group. *p<0.05, **p<0.01, ***p<0.001.

Next, we examined the effects of FTI-277 on burn-induced metabolic alterations. Consistent with previous studies [41], glycogen content in skeletal muscle of vehicle-treated burned mice was markedly decreased to 22% of that of vehicle-treated sham-burned mice (p<0.05). FTI-277 treatment restored glycogen content in burned mice to the level comparable to those in sham-burned mice (Fig. 5A).

To assess protein breakdown, we measured *ex vivo* release of amino acids by skeletal muscle during 2-h incubation. The amount of amino acids released by skeletal muscle of vehicle-treated burned mice was significantly increased to 392% of that of vehicle-treated sham-burned mice (p<0.001). FTI-277 treatment prevented burn-induced increased amino acids release compared with vehicle alone (p<0.001) (Fig. 5B).

*Ex vivo* lactate release by skeletal muscle of vehicle-treated burned mice was also markedly increased to 1,119% of that of vehicle-treated sham-burned mice (p<0.001). FTI-277 treatment significantly ameliorated burn-induced increased lactate release compared with vehicle alone (p<0.05) (Fig. 5C). Similarly, plasma lactate level of vehicle-treated burned mice was significantly increased to 280% of that of vehicle-treated sham-burned mice (p<0.01). Plasma lactate level of burned mice was attenuated by FTI-277 treatment to the level comparable to those of sham-burned mice (p<0.05) (Fig. 5D). Unlike increased triglycerides content in obesity-induced muscle insulin resistance [42], triglycerides content in skeletal muscle was not altered by burn or FTI-277 (Fig. 5E).

In contrast to the effects of FTI-277 in burned mice, FTI-277 did not significantly alter glycogen content, release of amino acids and lactate, and plasma lactate levels in sham-burned mice.

### Effects of FTI-277 on protein farnesylation in skeletal muscle of burned mice

**Figure 6:**
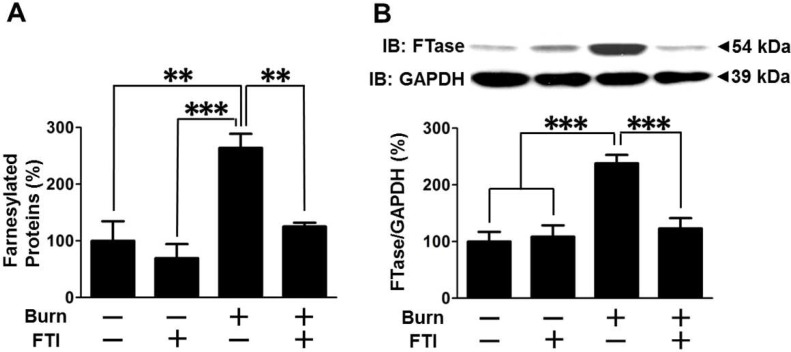
FTI-277 treatment prevented burn-induced increased protein farnesylation and FTase expression in skeletal muscle. (A) The amount of farnesylated proteins was increased in skeletal muscle at 3 days after burn compared with sham-burn. FTI-277 treatment prevented burn-induced increase in farnesylated proteins. n = 10 mice per group. (B) Burn increased farnesyltransferase (FTase) protein expression compared with sham-burn. FTI-277 treatment significantly inhibited burn-induced increased FTase expression. n = 6 mice per group. **p<0.01, ***p<0.001.

When treated with vehicle alone, burn significantly increased farnesylated proteins and FTase protein expression in muscle at 3 days after burn compared with sham-burn (Fig. 6). FTI-277 treatment reversed burn-induced increased farnesylated proteins compared with vehicle alone (p<0.01) (Fig. 6A). In sham-burned mice, there seems a trend of decreased farnesylated proteins by FTI-277, but there was no statistically significant difference between vehicle- and FTI-277-treated sham-burned mice. Of note, FTI-277 treatment reversed burn-induced increase in FTase expression (p<0.001) (Fig. 6B), although it did not alter FTase expression in sham-burned mice.

### Effects of FTI-277 on inflammatory gene expression in skeletal muscle of burned mice

**Figure 7:**
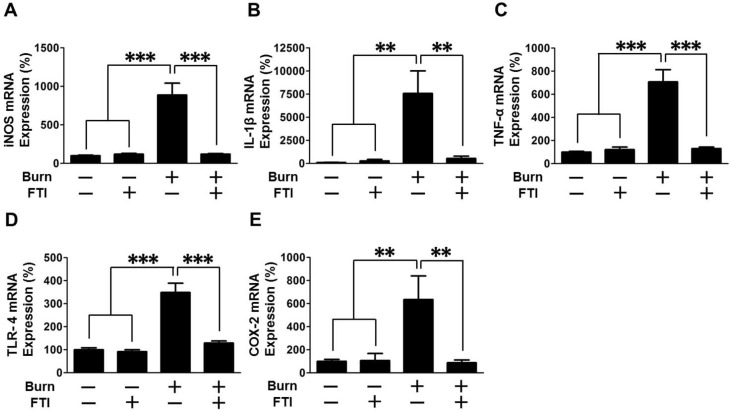
FTI-277 treatment reversed burn-induced induction of inflammatory genes expression in skeletal muscle. At 3 days after burn or sham-burn, mRNA expression of inflammatory genes was examined in skeletal muscle. mRNA levels of iNOS, IL-1β, TNF-α, TLR-4 and COX-2 were increased by burn. FTI-277 treatment prevented burn-induced induction of these genes. n = 8 mice per group. **p<0.01, ***p<0.001.

Inflammatory response plays a crucial role in obesity- and stress (e.g., burn)-induced insulin resistance [28]. As expected, mRNA levels of iNOS, IL-1β, TNF-α, TLR-4 and COX-2 were markedly increased after burn in vehicle-treated mice, all of which were reversed or significantly attenuated by FTI-277 treatment (Fig. 7). In sham-burned mice, FTI-277 did not alter mRNA expression of these genes.

### Effects of FTI-277 on circulating alarmins in burned mice

**Figure 8:**
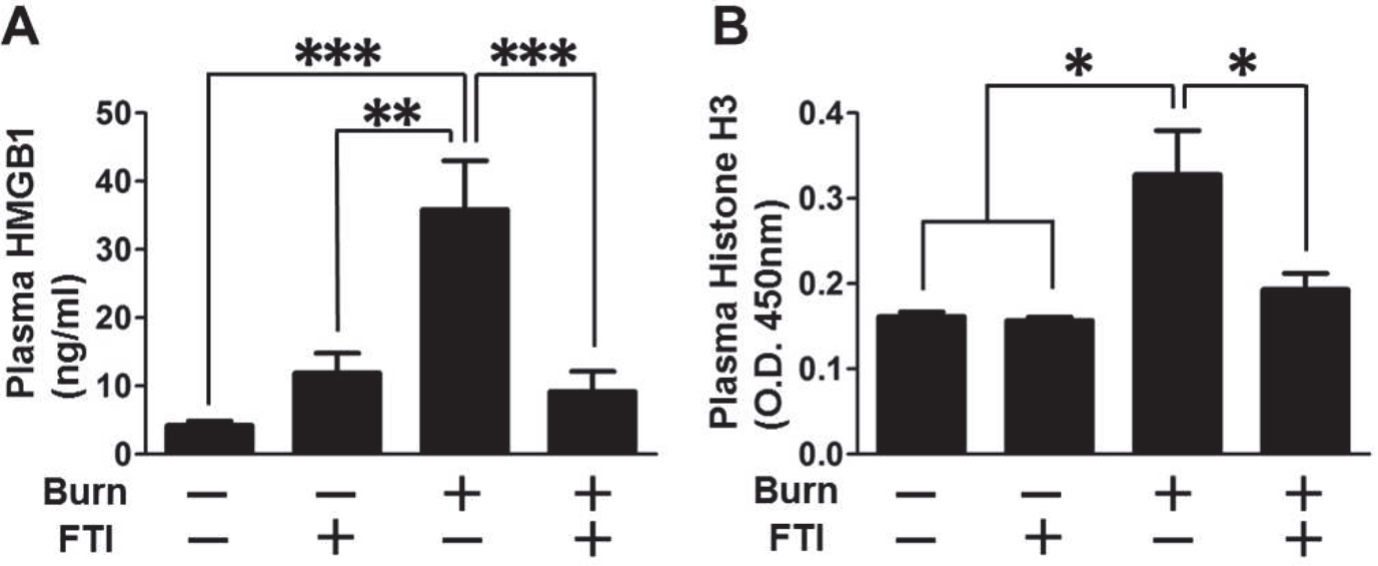
FTI-277 treatment prevented burn-induced increased circulating levels of HMGB1 and histone H3. (A) Plasma HMGB1 concentration was increased at 3 days after burn, which was prevented by FTI-277. **p<0.01, ***p<0.001, n = 8 mice per group. (B) Plasma histone H3 concentration was increased at 3 days after burn. FTI-277 attenuated burn-induced increase in histone H3 concentration. n = 4 mice per group for sham-burned mice, n = 9–11 mice per group for burned mice. *p<0.05.

Circulating alarmins (also known as endogenous damage-associated molecular patterns, DAMPs) such as HMGB1 [43] and histone H3 [44], have emerged as a major mediator of systemic inflammatory response. Consistent with elevated circulating HMGB1 concentration in burned rats [45] and burn patients [46] in previous studies, burn increased plasma HMGB1 and histone H3 levels, which were reversed by FTI-277 (Fig. 8).

## Discussion

Here, we show that burn-induced impaired muscle insulin signaling, attenuated insulin-stimulated glucose uptake in muscle and metabolic alterations were associated with increases in FTase expression and farnesylated proteins in mouse skeletal muscle, and that these burn-induced alterations were reversed or ameliorated by treatment with FTase inhibitor, FTI-277. These findings indicate that increased protein farnesylation plays a pivotal role in burn-induced insulin resistance and metabolic derangements in mouse skeletal muscle.

Consistent with our previous studies in rodents [31,32,41,47], burn injury resulted in: (1) attenuated insulin-stimulated phosphorylation of IR, IRS-1, Akt and GSK-3β (Figs. 2, 3); (2) decreased IRS-1 protein expression (Fig. 2E); and (3) increased PTEN protein expression (Figs. 1E, 4D). In addition, we found that burn increased PTP-1B expression in skeletal muscle (Figs. 1D, 4B). It is conceivable that decreased IRS-1 expression, and increased expression of PTP-1B and PTEN may contribute in concert to impair the IR-IRS-1-Akt-mediated insulin signaling in skeletal muscle of burned mice. Importantly, FTI-277 reversed attenuated insulin signaling along with reversal of altered expression of IRS-1, PTP-1B and PTEN in burned mice (Figs. 2–4).

Burn-induced impaired insulin signaling and its reversal by FTI-277 correlated with burn-induced metabolic aberrations and the reversal of them (Figs. 2–5). The metabolic alterations in burned mice include increased release of amino acids and lactate from muscle *ex vivo*, increased plasma lactate levels, and decreased glycogen content in muscle (Fig. 5). Skeletal muscle is a major source of circulating lactate [18,48]. Increased lactate and decreased glycogen content are major features of insulin resistance in skeletal muscle [49,50].

Of interest, the maximum effects of burn injury on PTP-1B and PTEN expression, and plasma lactate levels were observed at 3 days post-burn, when FTase expression was significantly increased (Fig. 1). The time-dependent increases in PTP-1B and PTEN expression are in accord with our previous findings that the maximum effect on insulin resistance is observed at 3 days post-burn [32]. These findings are also consistent with the notion that insulin resistance is a major component of metabolic derangements in burns.

Unexpectedly, we found that plasma lactate was not significantly increased within 24 h post-burn (Fig. 1E). Hyperlactatemia can result from the metabolic shift, which is referred to as cytopathic hypoxia or pseudohypoxia, where glycolytic ATP synthesis predominates over mitochondrial oxidative phosphorylation even under normoxic condition. In line with this, the *ex vivo* lactate release by muscle was markedly increased after burn although the muscle was incubated in the fully oxygenated buffer (Fig. 5C). These findings suggest that hyperlactatemia at 3 days post-burn may be attributable, at least in a significant part, to the metabolic shift rather than impaired microcirculation (hypoxia). Importantly, this is consistent with a previous study that altered glucose metabolism is a contributor to elevated plasma lactate concentration over and above deficit in oxygen availability in severely burned patients [51].

Of note, the protective effects of FTI-277 on insulin signaling and metabolic derangements were accompanied by reversal or mitigation of burn-induced induction of inflammatory genes in skeletal muscle and increased circulating alarmins (i.e., HMGB1 and histone H3) (Figs. 7, 8). These results are in line with previous studies that FTase inhibitors elicit anti-inflammatory action in non-immune cells under pathophysiological conditions [52–54]. Inflammation can be both adaptive and detrimental, but excessive inflammatory response is associated with the worse clinical outcome in many cases of major trauma, including burn injury [55]. Importantly, inflammatory response plays a critical role in insulin resistance [28–30]. We have previously shown that gene disruption of inducible nitric oxide synthase (iNOS), a major mediator of inflammation, inhibits burn and obesity-induced insulin resistance in mice [32,56]. FTI-277 prevented burn-induced iNOS expression (Fig. 7A). These data suggest that FTI-277-mediated reversal of induction of inflammatory genes, including iNOS, may play a role in prevention of burn-induced insulin resistance and metabolic alterations. It should be noted, however, that FTI-277 seems more efficacious in preventing burn-induced muscle insulin resistance compared with gene disruption of iNOS [32]. FTI-277 almost completely reversed impaired insulin signaling in skeletal muscle of burned mice to the level observed in sham-mice (Figs. 2–4). In contrast, iNOS deficiency significantly ameliorated insulin resistance, but not fully reversed it in our previous study in mice [32]. It is suggested that both iNOS-dependent and -independent mechanisms may be involved in the beneficial effects of FTI-277.

**Figure 9:**
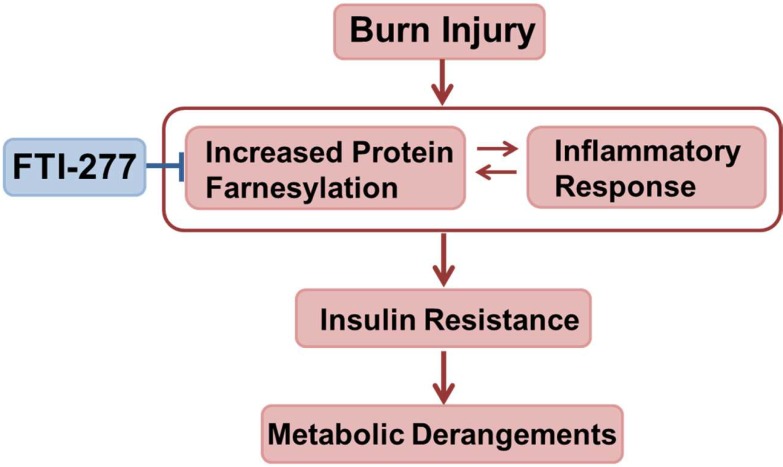
A possible role of protein farnesylation as a component of inflammatory response in burn-induced muscle insulin resistance and metabolic derangements. Our findings suggest that increased protein farnesylation may function as an upstream enhancer of burn-induced inflammatory response as well as a downstream mediator, forming a vicious cycle. This, in turn, may cause and/or exacerbate muscle insulin resistance and metabolic derangements. It is conceivable that FTI-277 may reverse burn-induced insulin resistance and metabolic dysfunction by controlling inflammatory response.

Burn injury increased FTase expression and farnesylated proteins in muscle (Figs. 1, 6). These findings are in accord with previous studies that LPS, interferon-γ, and sepsis increase farnesylated proteins [22,57,58]. It is suggested that increased protein farnesylation may be associated with inflammatory response, although little is known about the underlying mechanisms. It is noteworthy that treatment with FTI-277 reversed not only increased farnesylated proteins but also elevated expression of FTase in burned mice (Fig. 6), which paralleled attenuated inflammatory response (Figs. 7, 8). FTI-277 is an analogue of farnesyl pyrophosphate, the substrate of FTase, and thereby functions as a competitive inhibitor of FTase. FTI-277 is, however, not capable of directly modulating FTase expression. Hence, the reversal of the increased FTase expression by FTI-277 in burned mice cannot be accounted for by the direct effect of FTI-277. Collectively, it is conceivable that inflammation may increase expression and/or activity of FTase and that FTI-277 might reverse burn-induced increased FTase expression by attenuating inflammatory response. One can speculate, therefore, that increased farnesylation may function as a nodal point of inflammatory spiral by acting as an upstream enhancer of inflammation as well as a downstream mediator, thereby forming a vicious cycle, which, in turn, causes muscle insulin resistance and metabolic derangements in burns. Overall, our data suggest that FTI-277 may reverse insulin resistance and metabolic aberration by inhibiting burn-induced inflammatory spiral where increased protein farnesylation plays a role (Fig. 9). It should be noted, however, that FTI-277 is not a simple anti-inflammatory agent. Our previous study has shown that FTI-277 improves immune cell function in septic mice, whereas FTI-277 ameliorates systemic inflammatory response as indicated by the decrease in circulating HMGB1 concentration in septic mice [22]. It is possible, therefore, that the effects of FTI-277 on inflammatory response may differ dependent on cell types and the cellular context. Further studies are required to clarify this point.

Our date raise the possibility that farnesylation of some CAAX motif-containing proteins may be increased after burn, which, in turn, contributes to the burn-induced insulin resistance. However, targets proteins of burn- or inflammation-induced farnesylation are not known. We are currently in the process of identifying such FTase substrates that play a role in the burn-induced insulin resistance.

In conclusion, our data indicate that increased protein farnesylation plays an important role in burn-induced development of insulin resistance and related metabolic derangements (i.e., altered lactate and glycogen metabolism, increased protein breakdown) in mouse skeletal muscle as well as in inflammatory response post-burn injury. It is possible that inhibition of protein farnesylation may play a role in the pleiotropic beneficial effects of statins in burns. These findings identify FTase as a novel potential molecular target to reverse skeletal muscle insulin resistance and metabolic disturbance, and to control inflammatory response in burn patients.

## Supporting Information

S1 Fig Protein expression of GAPDH was not altered by burn, FTI-277 or insulin. (A) Burn injury did not alter GAPDH protein expression in skeletal muscle from 6 h through 7 days post-burn, as compared with naïve mice (Control). n = 4 per group. (B) Protein expression of GAPDH was not altered by burn, FTI-277 or insulin following on overnight fasting at 3 days post-burn or sham-burn. n = 5 per group for saline-injected mice, n = 6 per group for insulin-injected sham-burned mice, n = 8 per group for insulin-injected burned mice. (C) Protein expression of GAPDH was not altered by burn or FTI-277 following 4-h fasting at 3 days post-burn or sham-burn. n = 6 per group. NS: not significant.
(TIF)

S2 Fig FTI-277 treatment inhibited burn-induced increased phosphorylation of PTEN in skeletal muscle. At 3 days after burn or sham-burn, phosphorylation of PTEN at serine 380 was increased in vehicle-treated burned mice compared with sham-burned mice. FTI-277 treatment significantly decreased phosphorylated PTEN expression in burned, but not sham-burned, mice. n = 6 mice per group. ***p<0.001.
(TIF)
