# Supplementary figures and images for: Role of Protein Farnesylation in Burn-Induced Metabolic Derangements and Insulin Resistance in Mouse Skeletal Muscle
Source: PLoS One. 2015 Jan 16;10(1):e0116633. doi: 10.1371/journal.pone.0116633 (PMC4296934; doi:10.1371/journal.pone.0116633)

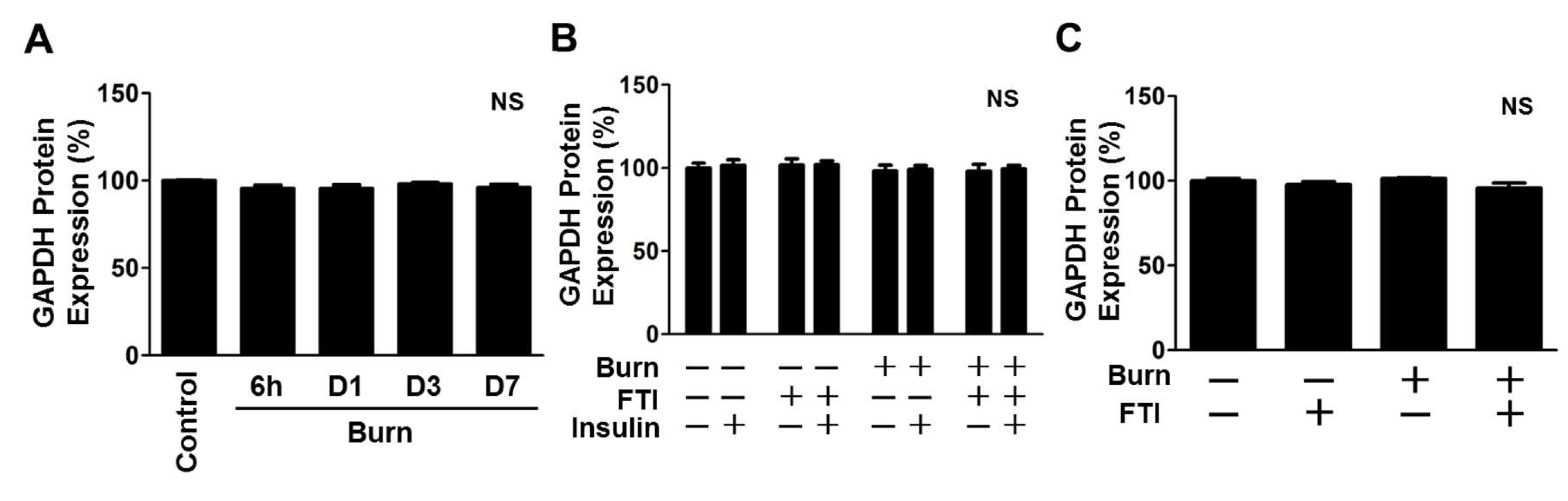

Supplement: S1 Fig — (A) Burn injury did not alter GAPDH protein expression in skeletal muscle from 6 h through 7 days post-burn, as compared with naïve mice (Control). n = 4 per group. (B) Protein expression of GAPDH was not altered by burn, FTI-277 or insulin following on overnight fasting at 3 days post-burn or sham-burn. n = 5 per group for saline-injected mice, n = 6 per group for insulin-injected sham-burned mice, n = 8 per group for insulin-injected burned mice. (C) Protein expression of GAPDH was not altered by burn or FTI-277 following 4-h fasting at 3 days post-burn or sham-burn. n = 6 per group. NS: not significant. (TIF) [file pone.0116633.s001.tif]

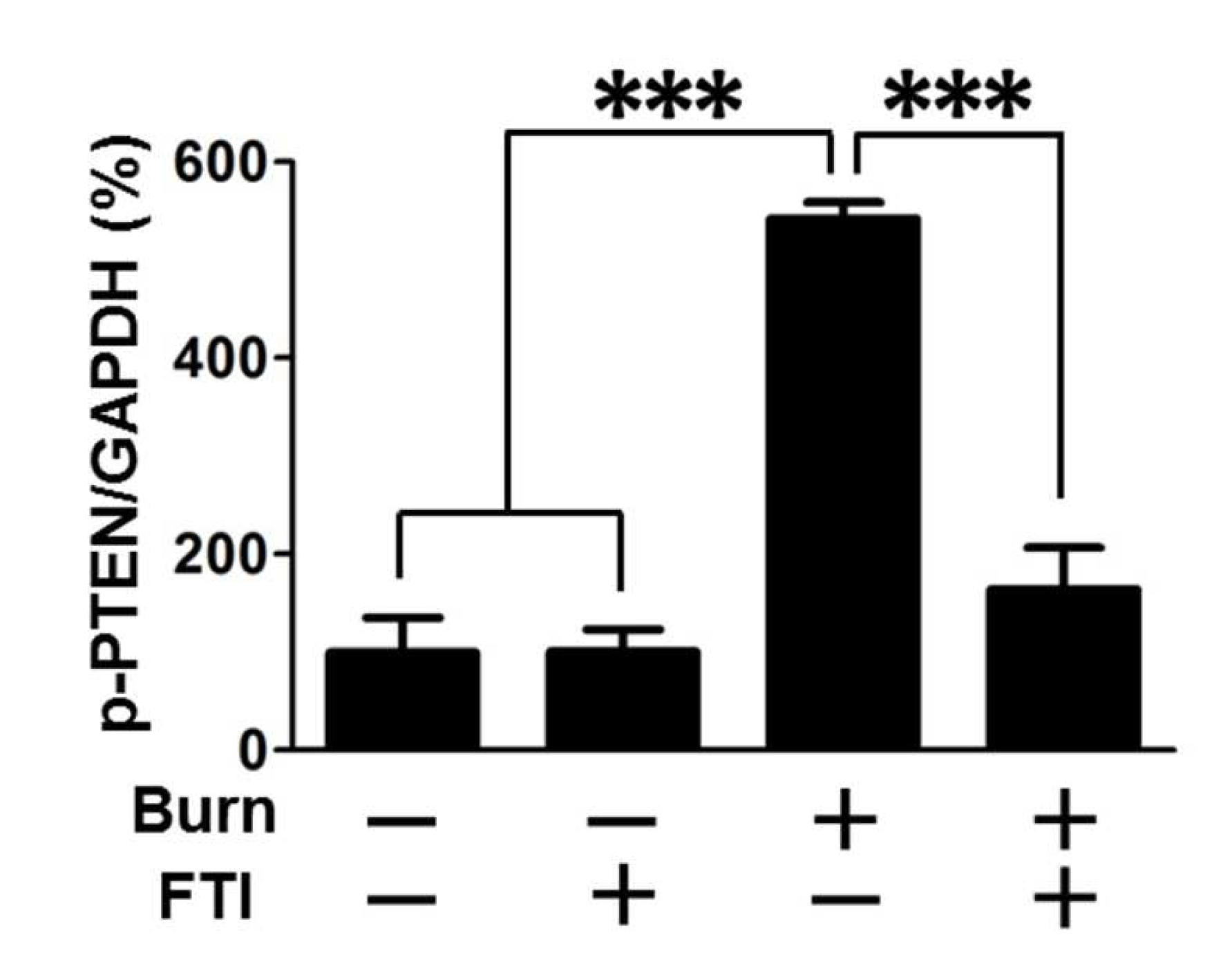

Supplement: S2 Fig — At 3 days after burn or sham-burn, phosphorylation of PTEN at serine 380 was increased in vehicle-treated burned mice compared with sham-burned mice. FTI-277 treatment significantly decreased phosphorylated PTEN expression in burned, but not sham-burned, mice. n = 6 mice per group. ***p<0.001. (TIF) [file pone.0116633.s002.tif]
